# Expression Profiles of Zebrafish (*Danio rerio*) Lysozymes and Preparation of c-Type Lysozyme with High Bacteriolytic Activity against *Vibrio vulnificus*

**DOI:** 10.3390/antibiotics11121803

**Published:** 2022-12-12

**Authors:** Hua Chen, Xi Chen, Tie-Ying Song, Jun-Qing Ge

**Affiliations:** 1 Institute of Biotechnology, Fujian Academy of Agricultural Sciences, Fuzhou 350003, China; 2 Fujian Province Key Laboratory of Special Aquatic Formula Feed (Fujian Tianma Science and Technology Group Co., Ltd.), Fuzhou 350308, China

**Keywords:** lysozyme, zebrafish, expression, *Vibrio vulnificus*, antimicrobial activity

## Abstract

Lysozyme is a crucial component of the host’s innate immune system. Due to its natural non-toxic and harmless characteristics, lysozyme is considered to be an ideal antibiotic substitute. In this study, we analyzed the expression profiles of lysozymes from zebrafish (*Danio rerio*) in uninfected or *V. vulnificus*-infected tissues using real-time quantitative PCR (qPCR). Furthermore, lysozymes that might be involved in the defense against *V. vulnificus* were selected for over-expression, and the antibacterial activity of over-expressed lysozyme proteins were evaluated using *V. vulnificus*. The results showed that three types of zebrafish lysozyme, i.e., c-type lysozyme (DrLysC), g1-type lysozyme (DrLysG1), and g2-type lysozyme (DrLysG2), were identified, and *V. vulnificus* infection significantly changed the expression levels of DrLysC and DrLysG1. Then, DrLysC and DrLysG1 were over-expressed in *E. coli*, and the purified recombinant DrLysC (rDrLysC) showed more potent antibacterial activity against *V. vulnificus*. This finding lays the foundation for further application of rDrLysC to treat *V. vulnificus* infection.

## 1. Introduction

As aquatic vertebrates, fish have a strong innate immune system, which plays an essential role in the early defense against pathogens [1]. Lysozyme, a crucial component of the innate immune system, exists ubiquitously in animals, plants, fungi, and bacteria [2]. Lysozyme can eliminate bacteria by destroying the β-1,4-glycosidic bond in peptidoglycan in the bacterial cell wall [3]. Based on differences in structural, catalytic, and immunological characteristics, lysozymes are generally classified into six types, i.e., chicken or conventional-type (c-type), invertebrate-type (i-type), goose-type lysozyme (g-type), T4 phage lysozyme, bacterial lysozyme, and plant lysozyme [4]. Until now, only c-type and g-type lysozymes have been identified in teleost fishes.

The c-type lysozyme was first isolated from rainbow trout (*Oncorhynchus mykiss*) [4], and later, it was also isolated from orange-spotted grouper (*Epinephelus coioides*) [5], Japanese flounder (*Paralichthys olivaceus*), Senegalese sole (*Solea senegalensis*) [6], and other fishes. Similarly, the teleost g-type lysozyme was first discovered from Japanese flounder (*P. olivaceus*) [7] and then from fishes including common carp (*Cyprinus carpio* L.) [8], mandarin fish (*Siniperca chuatsi*) [9], large yellow croaker (*Larimichthys crocea*) [10], Atlantic cod (*Gadus morhua L*) [11], and turbot (*Scophthalmus maximus*) [12]. Both c-type and g-type lysozymes are present in flounder (*P. olivaceus*), grass carp (*Ctenopharyngodon idella*), and turbot (*S. maximus*) [13]. Lysozyme is considered to be one of the important anti-bacterial molecules in fish [4] since it plays a vital role in host responses against bacterial infections [14]. In fish, lysozyme expression changes in different tissues to cope with pathogen infection [15].

Antibiotics are used to treat bacterial diseases; however, their continuous use has resulted in a variety of drug-resistant bacteria, the deposition of drug residues, and environmental pollution [16]. Thus, there is an urgent need of developing efficient, safe, and environment-friendly antibiotic alternatives for sustainable development of the aquaculture industry. Lysozyme has a different antibacterial mechanism than antibiotics, and thus, its application does not result in drug resistance in bacteria, which makes it an ideal antibiotic substitute [2]. Certain fish lysozymes have antibacterial effects to dissolve Gram-positive or Gram-negative bacteria. For instance, recombinant rock bream (*Oplegnathus fasciatus*) g-type lysozyme could inhibit Gram-negative bacteria *V. salmonicida* and Gram-positive bacteria *Listeria monocytogenes* [17], and recombinant orange-spotted grouper (*E. coioides*) c-type lysozyme could inhibit Gram-positive bacteria *Streptococcus iniae* and Gram-negative bacteria *V. alginolyticus* [5].

*Vibrio vulnificus* is an important zoonotic pathogen, which causes skin ulcers, traumas, gastroenteritis, and primary sepsis in humans and aquatic animals, resulting in a high fatality rate and significant losses in the breeding industry. *V. vulnificus* FJ03-X2 is highly virulent and pathogenic strain, and it was previously isolated from a diseased European eel (*Anguilla anguilla*) by our group. We observed that the strain could significantly alter the expression of c-type lysozyme in different zebrafish tissues [18], suggesting that lysozyme might play a role in the defense against *V. vulnificus* [4].

Zebrafish c-type lysozyme (DrLysC) was first cloned, which could be detected for expression in zebrafish macrophage cell line [19]. Wang Z et al. [20] confirmed that zebrafish lysozyme plays a pivotal role in the bacteriolytic activity in fertilized eggs. In addition, Irwin DM and Gong Z [21] found that zebrafish also had two g-type lysozymes. In this study, three zebrafish (*Danio rerio*) lysozymes, namely DrLysC, g1-type lysozyme (DrLysG1), and g2-type lysozyme (DrLysG2), were identified, and their expression profiles in natural or *V. vulnificus*-infected tissues were analyzed using real-time quantitative PCR (qPCR) analyses. These two lysozymes, DrLysC and DrLysG1, which might be involved in the defense against *V. vulnificus*, were selected for over-expression. Furthermore, the antibacterial activity of the purified recombinant lysozyme proteins was evaluated using *V. vulnificus*. The results indicated that DrLysC was expressed in response to *V. vulnificus* infection, and the over-expressed rDrLysC showed excellent antibacterial activity against *V. vulnificus*. This study lays the foundation for further research on fish lysozymes and further application of rDrLysC.

## 2. Results

### 2.1. Composition and Constitutive Expression of Zebrafish Lysozymes

Three zebrafish lysozymes, including one c-type (DrLysC) and two g-type (DrLysG1 and DrLysG2), were retrieved from ZFIN (Table 1). The sequence analysis of these lysozymes showed that DrlysC had a low molecular weight and four disulfide bonds, while DrlysG1 and DrLysG2 had two conserved cysteine residues and had no disulfide bond. Unlike DrLysC and DrLysG1, the signal peptide was absent in DrLysG2. These differences indicated that these three lysozymes might play different roles during bacterial infection.

**Figure 1:**
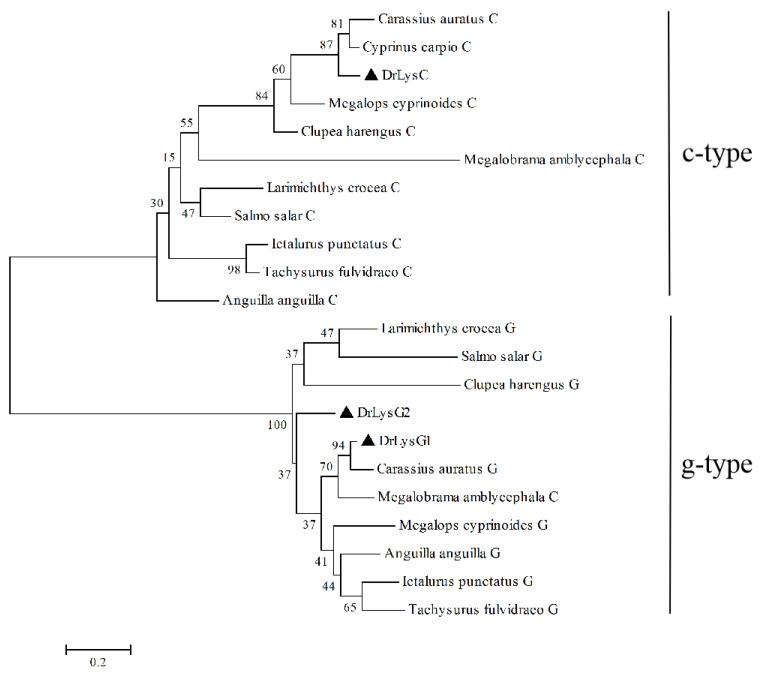
**Phylogenetic analysis of zebrafish lysozymes and their homologs in fish.** A phylogenetic tree was constructed by the neighbor-joining method using the Mega 4.0 software. The sequences of the lysozymes used in this analysis are as follows: DrLysC (NP_631919.1), *Carassius auratus* C (XP_026093809.1), *Cyprinus carpio* C (XP_018958317.1), *Anguilla anguilla* C (XP_035247404.1), *Larimichthys crocea* C (XP_019114157.2), *Megalobrama amblycephala* C (XP_048051439.1), *Clupea harengus* C (XP_012688691.1), *Megalops cyprinoides* C (XP_036378354.1), *Ictalurus punctatus* C (XP_017318497.1), *Tachysurus fulvidraco* C (XP_026996956.1), *Salmo salar* C (XP_014000972.1), DrLysG1 (NP_001002706.1), DrLysG2 (NP_001373416.1), *Carassius auratus* G (XP_026135699.1), *Anguilla anguilla* G (XP_035290251.1), *Larimichthys crocea* G (XP_010738712.1), *Megalobrama amblycephala* G (XP_048032707.1), *Clupea harengus* G (XP_031433526.1), *Megalops cyprinoides* G (XP_036404035.1), *Ictalurus punctatus* G (XP_017329515.1), *Tachysurus fulvidraco* G (XP_027010457.1), and *Salmo salar* G (XP_014031255.1). DrLysC, DrLysG1, and DrLysG2 are labeled by a triangle (▲). Numbers at nodes indicate bootstrap percentages (1000 replicates). The scale bar indicates evolutionary distance in base substitutions per site.

Furthermore, a phylogenetic tree was constructed based on the amino acid sequences of DrLysC, DrLysG1, DrLysG2 and their homologs in fishes (Figure 1). Fish lysozymes were clustered into two distinct branches, i.e., c-type and g-type [22]. DrLysC was clustered with Cypriniformes, Elopiformes, and Clupeiformes, while DrLysG1 showed the closest genetic distance with Cypriniformes then other fishes such as Anguilliformes, Elopiformes, Siluriformes, and Siluriformes. Moreover, only a few g2-type lysozymes were identified, so DrLysG2 was clustered into a separate branch.

**Figure 2:**
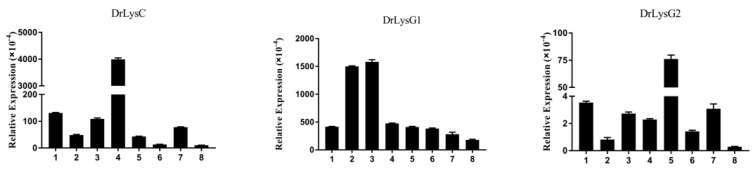
**Constitutive expression of DrLysC, DrLysG1, and DrLysG2 in different tissues of zebrafish**. Total RNA of different tissues of zebrafish was extracted for qPCR analysis. Relative expression of DrLysC, DrLysG1, and DrLysG2 was calculated using the 2^−∆∆Ct^ method, and β-actin was used as internal control. 1, heart; 2, liver; 3, spleen; 4, kidney; 5, gill; 6, intestine; 7, muscle; 8, skin.

To examine the constitutive expression patterns of DrLysC, DrLysG1, and DrLysG2, tissues of zebrafish were collected for qPCR analysis (Figure 2). The results showed that all three lysozymes were present in all the examined tissues. DrLysC had the highest expression level, and the expression of DrLysG2 was much lower than DrLysC and DrLysG1. In addition, DrLysC was highly expressed in the kidney, and DrLysG1 was highly expressed in the liver and spleen, while DrLysG2 was highly expressed in the gill.

### 2.2. Expression of the Lysozymes in V. vulnificus-Infected Zebrafish

**Figure 3:**
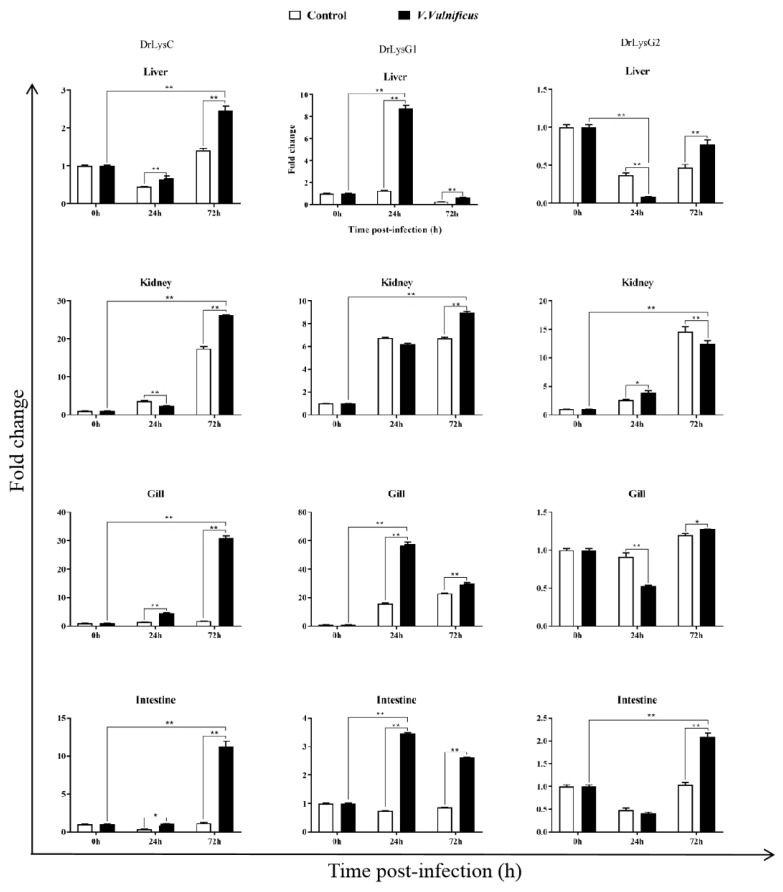
**The expression of DrLysC, DrLysG1, and DrLysG2 in different tissues of *V. vulnificus*-infected zebrafish.** Tissues of *V. vulnificus*-infected zebrafishes were collected at 0, 24, and 72 h post injection, and total RNA was extracted for qPCR analysis. Relative expression of DrLysC, DrLysG1, and DrLysG2 was calculated using the 2^−∆∆Ct^ method, and data are expressed as fold-change values. Each sample was tested in triplicate, and all data are presented as means ± SD. Statistical analysis was determined using two-way ANOVA for multiple comparisons. ** indicates highly significant differences (*p* < 0.001); * indicates significant differences (*p* < 0.05).

To understand the expression of the lysozymes in zebrafish, tissues of *V. vulnificus*-infected zebrafish were collected at 0, 24, and 72 h post infection (p.i.) for qPCR analysis. The results showed that DrLysC and DrLysG1 were up-regulated after *V. vulnificus* infection (Figure 3). The highest expression level of DrLysC was observed at 72 h p.i. and that of DrLysG1 at 24 h p.i., while the expression of DrLysG2 was much less changed. DrLysC expression in the kidney increased slightly at 24 h p.i. and peaked at 72 h p.i. However, DrLysG1 expression was significantly increased in the liver and peaked at 24 h p.i., which was significantly higher than the control group. These results indicated that DrLysC and DrLysG1 might contribute to the host’s innate immune defense upon exposure to *V. vulnificus*, while DrLysG2 may play different roles in different organs at different infection stages.

### 2.3. Expression of DrLysC and DrLysG1 in E. coli and Antimicrobial Activity Analysis

**Figure 4:**
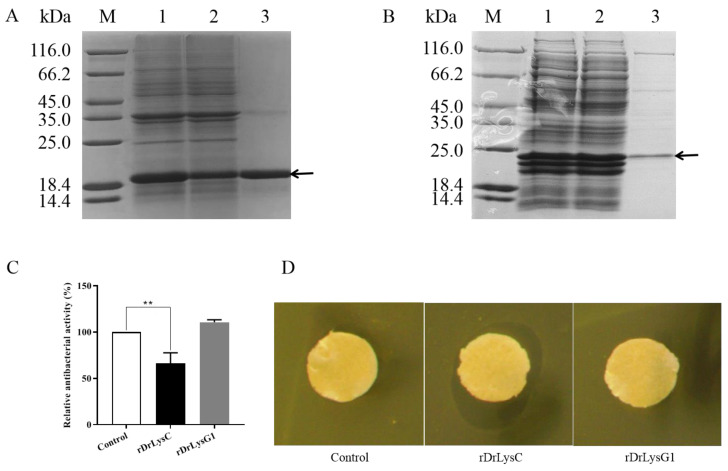
**Expression and antimicrobial activity determination of rDrLysC and rDrLysG1.** (**A**) Expression and purification of rDrLysC. M, protein marker; 1, induced bacterial lysate of pET-DrLysC/BL21; 2, residue of induced bacteria lysate of pET-DrLysC/BL21 after purification; 3, purified rDrLysC. (**B**) Expression and purification of rDrLysG1. M, protein marker; 1, induced bacterial lysate of pET-DrLysG1/BL21; 2, residue of induced bacteria lysate of pET-DrLysG1/BL21 after purification; 3, purified rDrLysG1. (**C**) Determination of the antimicrobial activity of the purified rDrLysC and rDrLysG1 against *V. vulnificus*. Relative antibacterial activity of the purified rDrLysC and rDrLysG1 were calculated using normal cultured *V. vulnificus* as control, and all samples were tested in triplicates. ** indicates highly significant differences. (**D**) Inhibition zones of the purified rDrLysC and rDrLysG1 against *V. vulnificus* strain FJ03-X2. The sterile filter paper containing 10 μg of rDrLysC or rDrLysG1, respectively, was evenly pasted on the plate, and filter paper containing sterile water was used as control. The plates were incubated at 28 °C overnight, then the inhibition zone was observed and photographed.

The sequences of DrLysC and DrLysG1 were optimized for expression in *E. coli*. SDS-PAGE analysis indicated that approximately 19.5 kDa and 24.2 kDa fusion proteins were highly expressed. Later, rDrLysC (Figure 4A) and rDrLysG1 (Figure 4B) were successfully purified using Ni-NTA Sefinose™ Resin, respectively. The antimicrobial activity of rDrLysC showed a significant inhibitory effect on *V. vulnificus*, and the inhibition rate was 66.14% (Figure 4C). However, rDrLysG1 showed no antimicrobial effect on *V. vulnificus*. In addition, agar diffusion analysis confirmed that the rDrLysC had an efficient inhibitory effect on *V. vulnificus*, while rDrLysG1 showed no inhibitory effect (Figure 4D).

## 3. Discussion

Only c-type and g-type lysozymes are identified from fishes, and most teleost fish contain both of them. The c-type lysozyme generally contains eight conserved cysteine residues (Cys) that form four pairs of the disulfide bond, while the number of Cys in the g-type lysozyme is usually uncertain, with no disulfide bonds [23]. Furthermore, c-type and g-type lysozyme genes of fish were less homologous, indicating their lesser biological diversity [23]. This study identified three lysozymes in zebrafish, including one c-type and two g-type lysozymes. Although two Cys residues were found in DrLysG1 and DrLysG2, disulfide bonds were not identified. It is probably because the two Cys residues might be not in the correct position to form a disulfide bond, and other structural stabilization mechanisms might exist in the g-type lysozyme of fish [23]. Further phylogenetic analysis indicated that c-type and g-type lysozymes in fish were clustered into two major branches, respectively. This indicated high homology of fish lysozymes and low homology between c-type and g-type lysozymes [22].

Lysozyme is a bactericidal, innate immune effector and one of the important evaluation indicators of host immune function. Both the c-type and g-type lysozymes could be detected in all of the examined tissues of Japanese eel (*A. japonica*), Chinese giant salamander (*Andrias davidianus*), and Qi river crucian carp (*Carassius auratus*), and the expression of g-type lysozyme in corresponding tissues was lower than that of c-type lysozyme [23]. In this study, three lysozymes, i.e., DrLysC, DrLysG1, and DrLysG2, were identified in all the examined zebrafish tissues, and they had different expression patterns in different tissues. This suggested that lysozymes might play different roles in different tissues and act synergistically to prevent the invasion of various pathogens. Moreover, the expression level of DrLysC was higher than DrLysG1 and DrLysG2. This indicated more potent antibacterial activity of DrLysC than that of DrLysG1 and DrLysG2 [24].

External stimulation could change lysozyme expression in fishes. The expression level of both DrLysC and DrLysG1 were up-regulated in *V. vulnificus*-challenged zebrafish, indicating that DrLysC and DrLysG1 might participate in the antibacterial response against *V. vulnificus*. Meanwhile, up-regulation of DrLysG1 peaked more quickly than that of DrLysC in the liver, gill, and intestine, while expression of DrLysG1 peaked at 24 h p.i. and then decreased gradually thereafter but was still higher than the control at 72 h p.i. This indicated that DrLysG1 might participate in bacterial clearance, which was also observed in *A. hydrophila*-infected Dabry’s sturgeon (*Acipenser dabryanus*) [24]. These results suggested that different zebrafish lysozymes might function synergistically and play a different roles in the antibacterial responses.

The function of lysozyme is determined primarily via its structure. Although fish lysozymes are more potent on Gram-negative bacteria, they also showed antibacterial activity against Gram-positive bacteria. For example, rainbow trout lysozyme had a significant inhibitory effect on Gram-negative bacteria, such as *V. anguillarum* and *Flavobacterium* sp. [25], while the expressed g-type lysozyme from turbot (*S. maximus*) showed strong antibacterial activity against Gram-positive bacteria *Micrococcus luteus* [12].

*V. vulnificus* is a bacterium that generally exists in the ocean, with a high mortality rate in immunocompromised patients [26]. Antibiotics are the main clinical drugs against *V. vulnificus* infection [26]. However, the increasing number of antibiotic-resistant *V. vulnificus* strains might have an adverse impact on public health [26]. Hence, the development of new antibiotic substitutes is important for *V. vulnificus* treatment. This study showed that both DrLysC and DrLysG1 play a crucial role in the defense against *V. vulnificus*. Over-expressed rDrLysC and rDrLysG1 with high purity (>90%) were successfully obtained. Further antimicrobial activity analysis showed that rDrLysC had 66.14% bacteriostatic activity against *V. vulnificus* but had no antimicrobial activity against *Micrococcus luteus*. On the contrary, rDrLysG1 showed no bacteriostatic activity against *V. vulnificus* but had antimicrobial activity against *Micrococcus luteus*. This suggested that zebrafish lysozymes might play different roles in the defense against different bacteria, and DrLysC might mainly act on Gram-negative bacteria and DrLysG1 mainly on Gram-positive bacteria. Further application study of the over-expressed rDrLysC paves a way for the prevention and treatment of *V. vulnificus* diseases.

## 4. Materials and Methods

### 4.1. Bacterial Strain and Culture Conditions

*V. vulnificus* strain FJ03-X2 was isolated from European eel by the Institute of Biotechnology, Fujian Academy of Agricultural Sciences [18], and cultured in Tryptic Soy Broth (TSB) medium at 28 °C without antibiotics. The strain FJ03-X2 was proved to be highly pathogenic to zebrafish.

### 4.2. Experimental Fishes

Wild-type zebrafishes (AB strain) were purchased from China Zebrafish Resource Center (CZRC) and cultivated in an aquatic animal culturing system at 28 °C with 0.05% salinity. Fishes were fed with a commercial pellet feed (SERA, Germany) twice a day, and the feeding amount was 1% of the body weight.

### 4.3. Sequence and Phylogenetic Analysis

The sequence of zebrafish lysozymes was retrieved from the Zebrafish Information Network (https://zfin.org/, accessed on 10 January 2020), and the conserved functional domains of the encoding proteins were analyzed using CDD Tools (https://www.ncbi.nlm.nih.gov/cdd/, accessed on 16 March 2020). The homologs of zebrafish lysozymes were retrieved from National Center for Biotechnology Information (https://www.ncbi.nlm.nih.gov/, 17 October 2022). A neighbor-joining phylogenetic tree was constructed using MEGA 4.0 software with 1000 bootstrap replicates.

### 4.4. Bacterial Challenge

Adult zebrafish (>6 months) with an average weight of 0.2 g were divided into two groups (120 fishes/group); one group was intraperitoneally injected (I.P.) with 10 μL of 2.94 × 10^5^ CFU/mL of *V. vulnificus* FJ03-X2 (LD_20_), and the other group was intraperitoneally injected with 10 μL of PBS and used as control. Fishes that died and those that showed apparent morbidity were removed and discarded.

### 4.5. Sample Collection

To examine constitutive gene expression patterns of zebrafish lysozymes, fishes were euthanized with tricaine methane sulfonate (MS-222, Sigma, St. Louis, MI, USA). Tissues including heart, liver, spleen, kidney, gill, intestine, muscle, and skin were collected from healthy fishes. Each sample was collected from 10 tails as a parallel group with three replications. To examine the response of lysozymes against *V. vulnificus* FJ03-X2, liver, kidney, gill, and intestine of *V. vulnificus*-infected fishes were collected at 0, 24, and 72 h post injection. All samples were collected from ten fishes and pooled together with three replicates.

### 4.6. RNA Extraction and qPCR Assay

Total RNA was extracted from collected samples using TRIzol reagent (Invitrogen, USA). Quality and concentration of the isolated RNA were determined via DeNovix DS-11 Spectrophotometer/Fluorometer and stored at −70 °C until further use. Further, 1 µg of total RNA was taken for cDNA synthesis using HiScri Ⅲ RT SuperMix for qPCR (+gDNA wiper) (Vazyme, Nanjing, China).

Specific primers were designed for the amplification of DrLysC, DrLysG1, and DrLysG2, and β-actin was used as the reference gene (Table 2). qPCR was conducted on a QuantStudio 3 system (ThermoFisher, Waltham, MA, USA) with ChamQ Universal SYBR qPCR Master Mix (Vazyme, Nangjing, China). Briefly, a 20 μL reaction volume containing 10.0 μL of 2 × ChamQ Universal SYBR qPCR Master Mix, 0.4 μL of each primer (10 μM), 2.0 μL of cDNA, and 7.2 μL of sterile water was prepared according to the manufacturer’s instructions. The amplification conditions were as follows: 95 °C for 30 s, followed by 40 cycles of 95 °C for 5 s and 60 °C for 34 s, followed by a melting curve analysis. Constitutive expression of zebrafish lysozymes was calculated using the 2^−∆∆Ct^ method, and β-actin was used as the internal control. Relative expression of zebrafish lysozymes in *V. vulnificus*-infected fishes was calculated using the 2^−∆∆Ct^ method, and data are expressed as fold-change values. Each sample was tested in triplicate, and all data are presented as means ± SD. Statistical analysis was performed using two-way ANOVA for multiple comparisons. *p* < 0.05 indicated a significant difference.

### 4.7. Expression and Purification of Recombinant Protein

The nucleotide sequence of DrLysC (GenBank accession No.: NM_139180.1) and DrLysG1 (GenBank accession No.: NM_001002706.1) were optimized according to their amino acid sequences. DrLysC and DrLysG1 were synthesized and cloned into pET-28a(+) expression vector. The constructed plasmids pET-DrLysC and pET-DrLysG1 were transformed into competent *E. coli* BL21 (DE3) cells, and protein expression was induced using IPTG. Expression of the recombinant protein was examined using SDS-PAGE, then the protein was purified using Ni-NTA Sefinose™ Resin (Sangon Biotech, Shanghai, China) and eluted using imidazole. The resulting recombinant DrLysC (rDrLysC) or DrLysG1 (rDrLysG1) was stored at −70 °C for further analysis.

### 4.8. Determination of the Antimicrobial Activity of Over-Expressed rDrLysC and rDrLysG1

A bacterial inhibition assay was performed as described previously [3] with minor modifications. Briefly, cultured *V. vulnificus* FJ03-X2 at the mid-logarithmic phase was collected and diluted to 1 × 10^6^ CFU/mL. Then, 50 μL bacterial suspension was taken and incubated with 10 μL (1μg/μL) purified rDrLysC or rDrLysG1 at 28 °C for 2 h, and sterile-water-incubated bacteria was used as control. The bacterial culture was added to a 96-well plate and cultured at 28 °C for 24 h, then OD_600_ was measured using an xMark™ Microplate Spectrophotometer (Bio-rad, USA). The growth of control bacteria was defined as 100%, and the relative antibacterial activity of the purified rDrLysC and rDrLysG1 were calculated. All the samples were tested in triplicate.

The inhibitory effect of the rDrLysC and rDrLysG1 on the growth of *V. vulnificus* strain FJ03-X2 was detected using the agar diffusion method. Briefly, the *V. vulnificus* was diluted to 10^6^ CFU/mL and coated on Tryptic Soy soybean Agar (TSA) solid plate. The sterile filter paper (5 mm diameter) containing 10 μg of rDrLysC or rDrLysG1, respectively, was evenly pasted on the plate, and sterile water was used as control. The plates were incubated at 28 °C for overnight, and then, the inhibition zone was observed and photographed.

## 5. Conclusions

Three lysozymes, namely DrLysC, DrLysG1, and DrLysG2, were identified in zebrafish. These lysozymes showed different expression patterns in all the examined zebrafish tissues. DrLysC and DrLysG1, which might participate in the defense against *V. vulnificus* were cloned and successfully expressed in *E. coli*. The expressed rDrLysC had efficient antibacterial activity against *V. vulnificus*. These results laid a foundation for further application of rDrLysC to treat *V. vulnificus* infection.

## Figures and Tables

**Table 1:** The characteristics of the sequence of zebrafish lysozymes.

Gene Symbol	Amino Acids	Molecular Weight (kD)	Signal Peptide	Conserved Cysteine Residue (Cys)	Disulfide Bond
DrLysC	151	17.1	Yes	8	4
DrLysG1	196	21.6	Yes	2	0
DrLysG2	191	21.1	No	2	0

**Table 2:** Primers used for the amplification of zebrafish lysozymes.

Gene Symbol	Forward Primer (5′-3′)	Reverse Primer (5′-3′)
β-actin	CACTTCACGCCGACTCAAAC	TCGGGGATGCTTATTTGCCA
DrLysC	GGCGTGGATGTCCTCGTGT	TCGGTGGGTCTTAAACCTGCT
DrLysG1	CTGGTAGGTGCGTGGGACA	GGGCAACAACATCATTAGCG
DrLysG2	CAAGTGTAAAATCTTCAAAGTTGCCA	TCGCCCCATCCTTTCAACA

## Data Availability

Not applicable.
